# The Long-Term Results of Three Catheter Ablation Methods in Patients With Paroxysmal Atrial Fibrillation: A 4-Year Follow-Up Study

**DOI:** 10.3389/fcvm.2021.719452

**Published:** 2021-10-14

**Authors:** Ling You, Xiaohong Zhang, Jing Yang, Lianxia Wang, Yan Zhang, Ruiqin Xie

**Affiliations:** Second Hospital of Hebei Medical University, Shijiazhuang, China

**Keywords:** catheter ablation, paroxysmal atrial fibrillation, recurrence, cryoablation, cryoablation plus radiofrequency ablation, radiofrequency ablation

## Abstract

**Aims:** Catheter ablation of paroxysmal atrial fibrillation (PAF) has been shown to be effective and safe. However, recurrence of PAF varies between 10 and 30% for radiofrequency ablation. There have been no reports comparing long-term recurrence rates following radiofrequency ablation, cryoablation, and three-dimensional guided cryoablation plus radiofrequency ablation. The aim of this study was to observe the long-term recurrence rate of PAF when treated by these three catheter ablation methods, and to explore clinical factors that can potentially predict PAF recurrence following catheter ablation.

**Methods:** There were 238 patients involved in this study, including 106 radiofrequency (RF) ablation cases (RF group), 66 cryoablation cases (Freeze group), and 66 cases treated by three-dimensional guided cryoablation combined with radiofrequency ablation (Freeze-plus-RF group). All patients underwent standardized follow-up. The recurrence rate of atrial fibrillation (AF) in the three groups was calculated. Predictive factors for the recurrence of AF were also investigated.

**Results:** At 48 months (the median follow-up period), the sinus rhythm maintenance rate was 77.4% in the RF group, 72.7% in the Freeze group, and 81.8% in the Freeze-plus-RF group. The maintenance rate of sinus rhythm was highest in the Freeze-plus-RF group, but differences among the three groups were not statistically significant. Further analysis found that the preoperative left atrial appendage emptying velocity (LAAEV) (recurrence vs. no recurrence, 56.58 ± 18.37 vs. 65.59 ± 18.83, respectively, *p* = 0.003), left atrial (LA) anteroposterior dimension (recurrence vs. no recurrence, 36.56 ± 4.65 vs. 35.00 ± 4.37, respectively; *p* = 0.028), and LA vertical dimension (recurrence vs. no recurrence, 56.31 ± 6.96 vs. 53.72 ± 6.52, respectively; *p* = 0.035) were related to postoperative recurrence. Multiple Cox regression analysis showed that only LAAEV was predictive of postoperative recurrence of PAF (hazard ratio: 0.979; 95% confidence interval: 0.961–0.997).

**Conclusion:** Our study found that there was no statistically significant difference in long-term recurrence rates among the RF, Freeze, and Freeze-plus-RF groups. Preoperative LAAEV is an independent predictor of postoperative recurrence of PAF.

## Introduction

Atrial fibrillation (AF) occurs in 0.71% of the Chinese population aged 35 years or older, and the incidence increases sharply with age (1). In patients who have AF even after medical treatment, catheter ablation is an alternative approach that can reduce complications and improve quality of life (2, 3).

Previous studies have confirmed the effectiveness and safety of catheter ablation in the treatment of paroxysmal atrial fibrillation (PAF) (4–6). Electrical isolation of the pulmonary vein (PV) is essential for catheter ablation of AF, which traditionally is achieved by radiofrequency catheter ablation (RCA) (7). However, in recent years a second method, cryoablation, has become a popular surgical approach for catheter ablation of atrial fibrillation (8). The PV must be treated for a shorter time with cryoablation than with RCA, and there are fewer postoperative complications (9, 10). Cryoablation produces a clear boundary, less thrombosis, and a lower incidence of cardiac perforation (11, 12).

However, due to the size and shape of the cryoablation equipment and the anatomical structure of the pulmonary vein, complete pulmonary vein isolation is in some cases difficult to achieve during cryoablation (13). We combined radiofrequency (RF) and cryoablation, called three-dimensional mapping guided cryoablation plus RF (Freeze plus RF), to achieve more perfect pulmonary vein isolation (14).

Recent studies have reported on the rate of recurrence of PAF 1–2 years following cryoablation and three-dimensional mapping-guided cryoablation (14), but the rate over longer periods has not been reported. This study aims to observe the long-term recurrence rate of these three approaches to catheter ablation in patients with PAF and explore potential clinical factors that can predict PAF recurrence after catheter ablation.

## Materials and Methods

### Study Population

This study involved 275 patients whose first catheter ablation of PAF was conducted in our center from March 2015 to March 2017. Among them, 238 patients were monitored for 4 years, and this cohort was divided into one of the three treatment groups: RCA, 106 patients; Freeze, 66; and Freeze plus RF, 66.

PAF is defined as spontaneous cessation of atrial fibrillation within 7 days of onset. Patient inclusion criteria included the following: (1) the ablation was the first for the patient, (2) there was no evidence of valvular heart disease, (3) there were more than two episodes of atrial fibrillation within the previous 6 months for which antiarrhythmic medication had been ineffective, and (4) the patient was followed up after the operation. Patients with atrial thrombus visible in transesophageal echocardiography were excluded.

All patients signed an informed consent form before their operation, and this study was approved by the ethics committee of the second hospital of Hebei Medical University.

### Catheter Ablation Procedure

#### Radiofrequency Ablation

Each patient underwent circumferential pulmonary vein isolation, with no additional ablation at extrapulmonary sites unless the patient was diagnosed with atrial flutter before the operation. In 3D electro-anatomical mapping (Carto 3, Biosense Webster), a mapping catheter is used to record pulmonary vein potentials before, during, and after circumferential pulmonary vein ablation (Lasso^®^ NAV eco, Biosense Webster). All patients in the radiofrequency group were treated using a 3.5-mm irrigated tip ablation catheter (SmartTouch, Biosense Webster). The operation was carried out for 20–30 min after blocking entrance and exit of pulmonary vein potentials. Pulmonary vein potential conduction was then monitored, and if there was recovery, ablation was continued. Tricuspid isthmus ablation was carried out for patients with a preoperative diagnosis of atrial flutter. All patients had CT scans of the pulmonary vein-left atrium before the operation to visualize the structure of the left atrium and pulmonary veins. Patients who experienced recurrence of atrial fibrillation during the first 3 months of postoperative follow-up were not considered for repeat radiofrequency ablation.

#### Cryoablation

Cryoablation was carried out with a single cryoballoon (Arctic Front Advance™ Cardiac CryoAblation Catheter, Medtronic, Minneapolis, MN) under fluoroscopy. The diameter of the cryoballoon was 28 mm or 23 mm according to the results of the pulmonary vein CT scan. The circular mapping catheter (Achieve, Medtronic) was first passed through the balloon catheter into the lumen of the pulmonary vein. The longest application of the first generation cryoballoon was 240 s and the lowest temperature was −60°C, while the longest application of the second generation cryoballoon was 180 s and the lowest temperature was −55°C. If two applications of the cryoablation were unsuccessful, additional cryoablation treatments could be carried out until complete electrical isolation of the pulmonary veins and bidirectional block of electrical conduction between the pulmonary veins and left atrium were achieved. No patients had any residual PV connection after acute application of the cryoballoon. If the patient had atrial flutter during freezing, additional radiofrequency ablation was applied.

Among the 66 patients in the Freeze group, 24 (36.4%) used the first generation cryoballoon. Before carrying out right pulmonary vein ablation, a secondary catheter was placed into the superior vena cava. During cryoablation, a cycle time of 999 ms was used to make the right phrenic nerve pulsate. If diaphragm movement was reduced or became undetectable, cryoablation was immediately terminated.

#### Cryoablation Combined With Radiofrequency Ablation

Cryoballoon ablation combined with RF ablation was used under the guidance of the EnSite NavX 3D mapping system. A circular mapping catheter (Achieve, Medtronic) was used to construct the configuration and build the structures of the pulmonary vein. The cryoballoon was positioned during cryoablation according to the results of the 3D mapping. At least 2 cryoablations were conducted for each pulmonary vein. If there was still electrical conduction in the pulmonary veins after two cryoablations, radiofrequency ablation could be used at additional locations, and complete electrical isolation of the pulmonary veins and bidirectional block of electrical conduction between the pulmonary veins and left atrium could finally be achieved. Among the 66 patients in the Freeze-plus-RF group, 7 (10.6%) used the first generation cryoballoon. An additional RF ablation was required to achieve pulmonary vein isolation in 9 patients. If the patient had atrial flutter during freezing, additional RF ablation was applied.

### Patient Follow-Up

In this study, patients were followed up weekly in the first month and then visited at 2, 3, 6, 9, and 12 months after discharge. The patients were then followed up once a year. ECGs, 24-h Holter records, and echocardiography images were examined at all visits. Patients with any palpitation discomfort during this period could come to the hospital at any time. In this study, recurrence of atrial fibrillation was defined by an ECG or 24-h ECG record of atrial fibrillation, atrial flutter, or atrial tachycardia lasting more than 30 s.

### Echocardiographic Examination

Examinations of real-time 3D ultrasound, 2D ultrasound, and transesophageal and Doppler echocardiography were carried out to measure left atrial size and function in all patients using a cardiac ultrasound device (iE33 machine equipped with X3-1 and X7-2t, Philips Medical Systems, Eindhoven, the Netherlands). Ultrasound parameters were measured under sinus rhythm in all patients. Left atrial (LA) dimensions were measured in the parasternal long axis view using 2D methods. LAMax refers to left atrial volume at the end of systole before opening of the mitral valve, while LAMin refers to the end of diastole before closing of the mitral valve. All patients underwent preoperative transesophageal echocardiography to obtain left atrial appendage emptying velocity (LAAEV). Left atrial appendage images were obtained from the base of the heart with the probe rotated by 0°, 45°, 90°, and 180°. LAAEV was measured using 1 representative value when rhythm was stable, or by averaging the value of 5 consecutive sinus waves when rhythm was variable due to respiration.

### Statistical Analysis

- Continuous variables are presented as mean ± standard deviation, and categorical variables as percentage with counts. Differences in continuous data between groups were compared using ANOVA. Chi-square tests or Fisher's exact tests were used for categorical variables. Receiver operating characteristic analysis was performed to determine the optimal cut-off value for the LAAEV in predicting AF recurrence after a single procedure. Survival curves were generated by Kaplan-Meier analysis.
- To evaluate predictors for recurrence of AF, Cox regression analysis was performed. All the predictors were evaluated by univariate Cox regression. Factors that were statistically significant in the univariate model were further investigated using the multiple Cox regression model.
- *P* < 0.05 was considered statistically significant. SPSS software (version 26.0, Chicago, IL) was used.

## Results

### Patients

There were 238 patients enrolled in this study, including 106 patients in the RF group (mean age 58.05 ± 10.04 years), 66 patients in the Freeze group (mean age 59.2 ± 11.89 years), and 66 patients in the Freeze-plus-RF group (mean age 61.68 ± 11.57 years). Baseline demographics of the patients are shown in Table 1. Except for the low proportion of hypertensive patients in the RF group, there were no statistically significant differences between the three groups. The operation achieved complete pulmonary vein isolation for all patients. One patient developed pericardial effusion 2 h after the operation and recovered after pericardiocentesis. During follow-up, one patient developed gastrointestinal bleeding 3 months after surgery. There were no operation-related deaths, although two patients died of gastric cancer, and one died in an accident.

**Table 1:** Patient characteristics at baseline before ablation.

	**RF (106 cases)**	**Freeze** **(66 cases)**	**Freeze plus RF (66 cases)**	***P*-value**
Age, years	58.05 ± 10.04	59.20 ± 11.89	61.68 ± 11.57	0.110
Male sex, n (%)	64 (60.4)	35 (53.0)	41 (62.1)	0.518
Diabetes, n (%)	13 (12.3)	10 (15.2)	14 (21.2)	0.288
Hypertension, n (%)	48 (45.3)	40 (57.6)	38 (57.6)	0.001
SBP, mmHg	133.98 ± 19.73	130.45 ± 15.72	135.21 ± 18.58	0.297
DBP, mmHg	83.10 ± 14.29	81.56 ± 11.97	82.17 ± 11.29	0.738
Heart rate, beats per minute	73.47 ± 15.27	75.77 ± 16.83	72.94 ± 16.22	0.443
Heart failure, n (%)	5 (4.7)	4 (6.1)	6 (9.1)	0.515
History of CAD, n (%)	1 (0.9)	1 (1.5)	2 (3.0)	0.581
Smoking, n (%)	15 (14.2)	9 (13.6)	14 (21.2)	0.390
Alcohol, n (%)	12 (11.3)	6 (9.1)	13 (19.7)	0.152
Phrenic nerve injury, n (%)	0	1 (1.5)	1 (1.5)	0.509
Vascular injuries (%)	2 (1.8)	1 (1.5)	1 (1.5)	0.216
Gastrointestinal bleeding, n (%)	1 (0.9)	0	0	0.458
Pericardial tamponade, n (%)	1 (0.9)	0	0	0.458

### Clinical Outcomes After a Single Procedure

**Figure 1:**
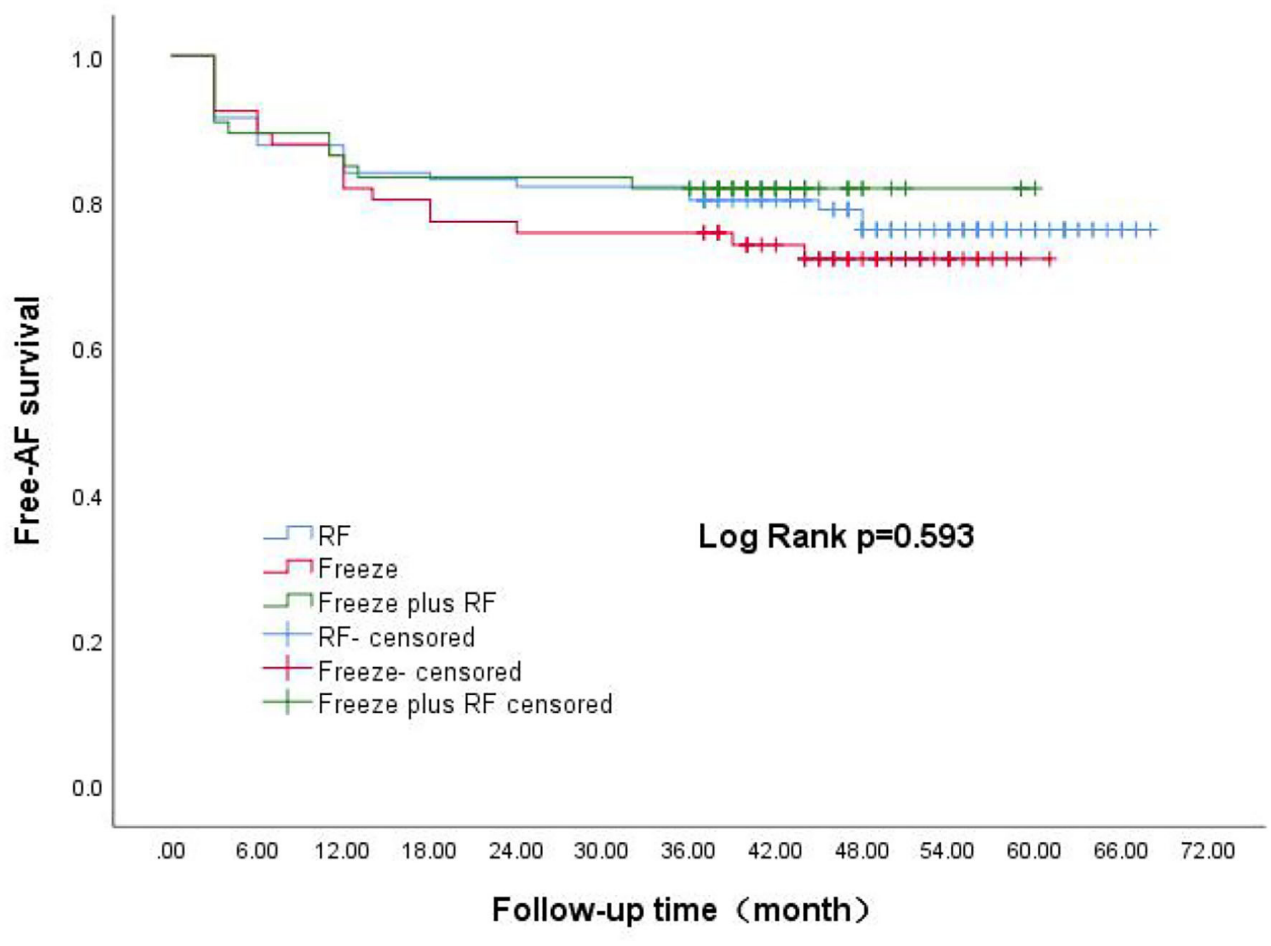
Kaplan-Meier analysis of AF-free rate after three different surgical procedures. AF, atrial fibrillation.

Survival curves for the three groups are shown in Figure 1. The median follow-up was 48 months for all three groups. The recurrence rates at 1, 2, 3, and 4 years for the RF group were 15.1, 17.9, 19.8, and 22.6%, respectively. The corresponding recurrence rates were 18.2, 22.7, 24.2, and 27.3% for the Freeze group and 16.8, 16.8, 18.2, and 18.2% for the Freeze-plus-RF group. Our study found that most recurrence of AF occurred in the first year after AF surgery, which accounted for 70.7% (RF), 66.7% (Freeze), and 81.8% (Freeze plus RF) of all recurrences (*P* = 0.234, Table 2). At 4 years, the maintenance rate of sinus rhythm was highest in the Freeze-plus-RF group (81.8%), but this was not significantly different from the RF (77.4%) and Freeze (72.7%) groups (*P* = 0.593). This may be due to the combination of the two ablation techniques used to achieve more complete pulmonary vein isolation. Of the recurrent patients, there was one case of atrial flutter in the Freeze group, and one case each of atrial flutter and atrial tachycardia in the Freeze-plus-RF group. All other atrial arrhythmias were atrial fibrillation.

**Table 2:** Sinus rhythm after different periods of follow-up in the 3 groups.

	**3 months**	**6 months**	**12 months**	**24 months**	**36 months**	**48 months**
RF	97 (91.5%)	93 (87.7%)	90 (84.9%)	87 (82.1%)	85 (80.2%)	82 (77.4%)
Freeze	61 (92.4%)	59 (89.4%)	54 (81.8%)	51 (77.3%)	50 (75.8%)	48 (72.7%)
Freeze 3D	60 (90.9%)	59 (89.4%)	55 (83.3%)	55 (83.3%)	54 (81.8%)	54 (81.8%)
*p*	0.951	0.923	0.866	0.634	0.666	0.460

### Factors Associated With Recurrence of Atrial Fibrillation After Catheter Ablation

**Figure 2:**
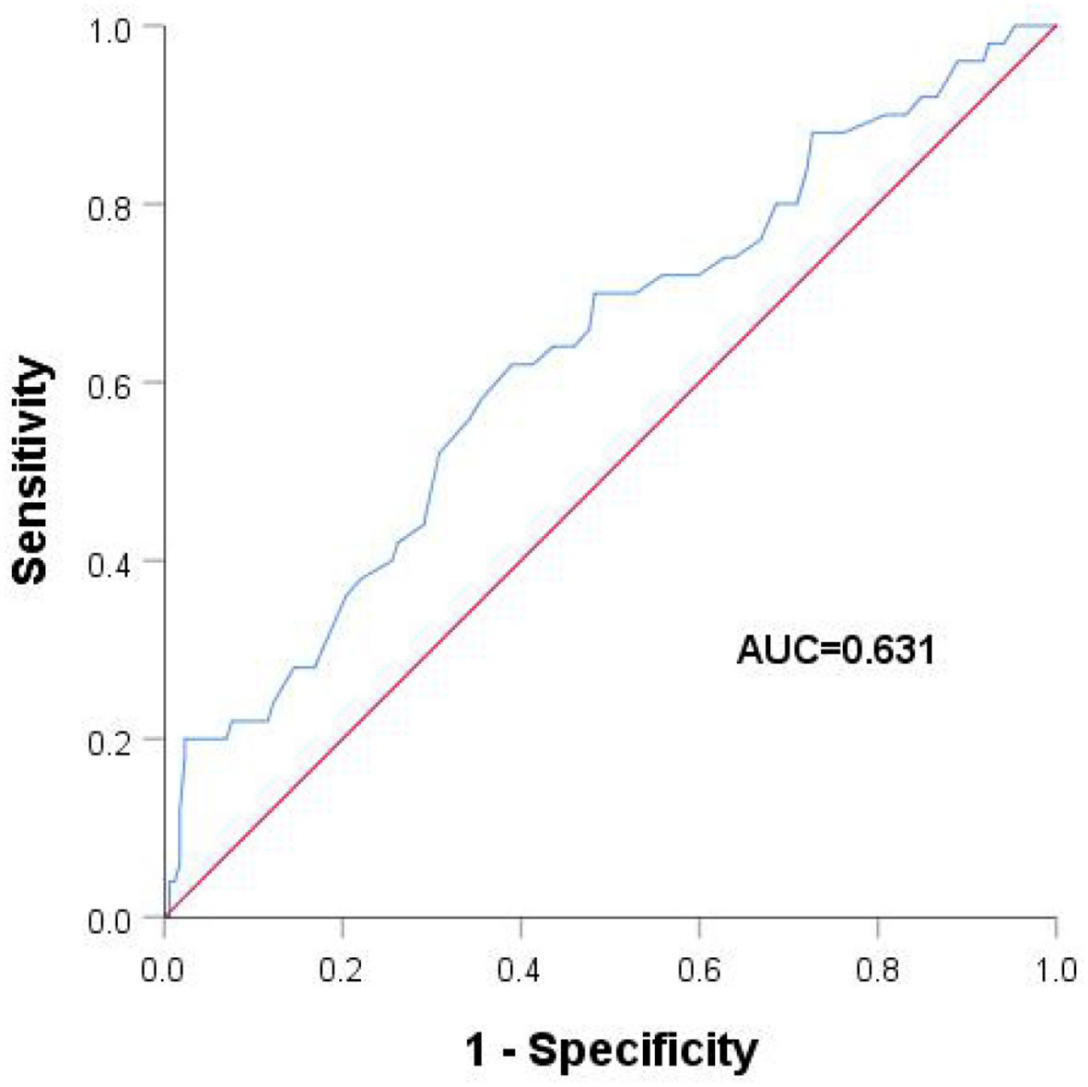
Receiver operating characteristic analysis with different left atrial appendage emptying velocities.

LAAEV was significantly lower in recurrent than in non-recurrent patients (56.58 ± 18.37 vs. 65.59 ± 18.83, respectively; *p* = 0.003). As shown in Table 3, the LA anteroposterior and vertical dimensions in recurrent patients (36.56 ± 4.65 and 56.31 ± 6.96, respectively) were both larger than in non-recurrent patients (35.00 ± 4.37 and 53.72 ± 6.52, respectively) (*p* = 0.028 and *p* = 0.035, respectively, for the two LA dimensions). All factors were further analyzed by the Cox regression model. Patients with higher LAAEV had a lower risk of recurrence of PAF (hazard ratio: 0.979; 95% confidence interval: 0.961–0.997) according to multivariate Cox regression (Table 4). Receiver operating characteristic curve analysis showed that the optimal cut-off value for LAAEV was 59.5 cm/s for predicting late recurrence of AF after a single procedure with a sensitivity of 62% and specificity of 61%. The area under the curve was 0.631 (*P* = 0.005; Figure 2).

**Table 3:** Characteristics of recurrence and no-recurrence patients.

	**All patients**	**Recurrence**	**No recurrence**	***P*-value (recurrence vs. no recurrence)**
Age, years	59.37 ± 11.06	57.30 ± 10.32	59.98 ± 11.22	0.117
Male, n (%)	130 (54.6)	26 (48.1)	114 (62.0)	0.070
Diabetes, n (%)	37 (15.6)	11 (20.4)	26 (14.1)	0.266
Hypertension n (%)	126 (52.9)	29 (53.7)	97 (52.7)	0.898
SBP (mm Hg)	133.34 ± 18.39	131.39 ± 22.11	133.91 ± 17.17	0.376
DBP (mm Hg)	82.41 ± 12.84	82.54 ± 15.66	82.37 ± 11.94	0.934
Heart rate, beats per minute	73.96 ± 15.96	74.04 ± 15.61	73.94 ± 16.10	0.969
Smoking, n (%)	44 (18.5)	9 (16.7)	35 (19.0)	0.695
Alcohol, n (%)	31 (13.0)	6 (11.1)	25 (13.6)	0.635
History of CAD, n (%)	4 (1.6)	1 (1.8)	3 (1.6)	0.911
Heart failure, n (%)	14 (5.9)	6 (11.1)	8 (4.4)	0.065
LVEF (%)	55.91 ± 16.52	57.59 ± 16.41	55.35 ± 16.57	0.421
LA anteroposterior dimension (mm)	35.37 ± 4.48	36.56 ± 4.65	35.00 ± 4.37	0.028
LA transverse dimension (mm)	38.35 ± 6.95	38.76 ± 4.59	38.21 ± 7.58	0.688
LA vertical dimension (mm)	54.34 ± 6.69	56.31 ± 6.96	53.72 ± 6.52	0.035
LAAEV (cm/s)	63.56 ± 19.07	56.58 ± 18.37	65.59 ± 18.83	0.003
LAVmax (mL/m^2^)	55.67 ± 17.19	59.72 ± 15.40	54.37 ± 17.60	0.145
LAVmin (mL/m^2^)	27.66 ± 12.40	30.97 ± 11.65	26.60 ± 12.51	0.099

*SBP, systolic blood pressure; DBP, diastolic blood pressure; LVEF, left ventricular ejection fraction; LAAEV, left atrial appendage emptying velocity; LAVmax, left atrial maximum volume index; LAVmin, left atrial minimum volume index*.

**Table 4:** Univariate and multivariate Cox regression analysis to recognize predictors of AF recurrence after a single procedure.

	**Univariate**			**Multivariate**		
**Variables**	**HR**	**CI**	***p***	**OR**	**CI**	***p***
Age, years	0.984	0.962–1.006	0.144			
Male, n (%)	0.619	0.363–1.056	0.079			
SBP (mm Hg)	0.994	0.979–1.009	0.420			
DBP (mm Hg)	1.000	0.979–1.021	0.988			
Heart rate, beats per minute	1.000	0.983–1.017	0.998			
Smoking, n (%)	0.881	0.431–1.802	0.728			
Alcohol, n (%)	0.846	0.362–1.978	0.700			
Hypertension, n (%)	1.056	0.618–1.804	0.841			
Diabetes, n (%)	1.510	0.779–2.930	0.222			
History of CAD, n (%)	1.153	0.159–8.350	0.888			
Heart failure, n (%)	2.148	0.919–5.020	0.078			
LVEF (%)	1.008	0.990–1.026	0.378			
LAAEV (cm/s)	0.976	0.961–0.992	0.003	0.979	0.961–0.997	0.023
LA anteroposterior dimension (mm)	1.066	1.004–1.132	0.037	1.049	0.963–1.143	0.276
LA vertical dimension (mm)	1.053	1.003–1.105	0.038	1.029	0.971–1.090	0.335
LA transverse dimension (mm)	1.008	0.964–1.054	0.740			
LAVmax (ml/m^2^)	1.014	0.995–1.034	0.154			
LAVmin (ml/m^2^)	1.023	0.996–1.050	0.094			

*SBP, systolic blood pressure; DBP, diastolic blood pressure; LVEF, left ventricular ejection fraction; LAAEV, left atrial appendage emptying velocity; LAVmax, left atrial maximum volume index; LAVmin, left atrial minimum volume index*.

## Discussion

This is the first study to compare long-term recurrence of PAF among patients treated by radiofrequency ablation, cryoablation, or cryoablation plus RF. More than half the recurrences in each of the three groups occurred in the first year after surgery. The study also found that LAAEV was a predictor of late recurrence after ablation.

Our study found that there were no differences in long-term recurrence among the RF ablation, cryoablation, and cryoablation-plus-RF groups. Previous studies have reported no difference in recurrence rate between radiofrequency ablation and cryoablation (10, 12, 15–17), which is consistent with our results. A meta-analysis reported by Maltoni et al. (10) showed no significant difference in efficacy between radiofrequency ablation and cryoablation in avoiding recurrence of atrial arrhythmia in patients with paroxysmal atrial fibrillation. Some studies have used radiofrequency ablation for supplementary treatment when complete pulmonary vein isolation was not achieved during cryoablation (15, 18), however, there was no comparison of recurrence between RF, cryoablation, and cryoablation supplemented by RF ablation. Kettering et al. (13) used both cryoballoon and radiofrequency ablation during secondary surgery on patients with recurrence following cryoballoon ablation. In their study, radiofrequency ablation was safer and more effective for patients with recurrence after cryoballoon ablation.

Several risk factors have been reported to be associated with recurrence of AF after ablation, such as hypertension and LA size and volume, among others (19–21). Two studies have found that a decrease in LAAEV is a predictor of recurrence of atrial fibrillation 1 year after radiofrequency ablation (22, 23). A decrease in LAAEV can also be used as a predictor of cardioversion in patients with nonvalvular AF (24–26). Our study found that high LAAEV was associated with a lower rate of AF recurrence. LAAEV is an important indicator of left atrial function (27). During development of atrial fibrillation, structural remodeling, atrial fibrosis and abnormal formation of atrial matrix will occur, resulting in decline of the left atrial function. These mechanisms are also the basis of AF recurrence after both drug and surgical cardioversion (28).

Wang et al. (6) reported that more than two-thirds of AF recurrence were in the first year after single or multiple procedures. Other studies have also shown that the rate of early recurrence of AF (ERAF, defined as AF recurrence within a 3-month blanking period) ranged from approximately 38.2–58.6% after a single ablation, and that ERAF significantly predicted late recurrence of AF (29–33). The mechanism of ERAF is unclear but is generally considered to involve acute thermal injury and an inflammatory response caused by radiofrequency energy and a transient reversible process (34, 35). In our study, we found that more than half of all recurrences were in the first year after a single operation. In addition to the above factors, others such as acute thermal injury and inflammatory response caused by catheter ablation may contribute to recurrence of AF. In addition, recovery of electrical connectivity between the pulmonary vein and left atrium, as well as trigger foci outside the pulmonary vein, may be more likely to occur 1 year after AF surgery.

Patients in this study came from a single center, which limited data variability. Both first and second generation cryoballoons were used with the Freeze and the Freeze-plus-RF groups, and there may have been differences in surgical outcomes using the two types of balloons. No patients had implanted devices that allowed continuous rhythm monitoring, and so some recurrences may have gone undetected. The main limitation of this article is that it was a non-randomized observational study, introducing potential bias which may have confounded the results.

## Conclusion

Our study found that long-term recurrence rates were not significantly different between the three surgical methods. The first year after the operation had the highest rate of recurrence following catheter ablation. Left atrial appendage emptying velocity is a predictor of long-term recurrence of paroxysmal atrial fibrillation after a single operation.

## Data Availability Statement

The original contributions presented in the study are included in the article/supplementary material, further inquiries can be directed to the corresponding author.

## Ethics Statement

The studies involving human participants were reviewed and approved by the Second Hospital of Hebei Medical University. The patients/participants provided their written informed consent to participate in this study. Written informed consent was obtained from the individual(s) for the publication of any potentially identifiable images or data included in this article.

## Author Contributions

LY is the first author responsible for thesis writing and data statistics. XZ, JY, LW, and YZ were responsible for data collection and follow-up. RX is responsible for the design of research ideas. All authors contributed to the article and approved the submitted version.

## Conflict of Interest

The authors declare that the research was conducted in the absence of any commercial or financial relationships that could be construed as a potential conflict of interest.

## Publisher's Note

All claims expressed in this article are solely those of the authors and do not necessarily represent those of their affiliated organizations, or those of the publisher, the editors and the reviewers. Any product that may be evaluated in this article, or claim that may be made by its manufacturer, is not guaranteed or endorsed by the publisher.
